# Supplementary material for: Supported storytelling through the ‘Life Threads’ approach for family members after traumatic brain injury: “We’ve been through all of this trauma, and you’re giving me some string?”
Source: PLoS One. 2026 May 18;21(5):e0349304. doi: 10.1371/journal.pone.0349304 (PMC13183248; doi:10.1371/journal.pone.0349304)
Supplement: S1 Table — This is a completed checklist to ensure reflexive TA was conducted rigorously. (DOCX) [file pone.0349304.s001.docx]

**Supplementary Material Table 1: Response to Braun and Clarke’s (2022) 15-point checklist for ‘good’ reflexive TA.**

| Process | No. | Criteria | Response |
| --- | --- | --- | --- |
| Transcription | 1 | The data have been transcribed to an appropriate level of detail; all transcripts have been checked against the original recordings for ‘accuracy’. | Yes (line 182) |
| Coding | 2 | Each data item has been given equal attention in the coding process. | Yes |
|  | 3 | Themes have not been generated from a few vivid examples (an anecdotal approach), but instead the coding process has been thorough, inclusive and comprehensive. | Yes |
|  | 4 | All relevant extracts for all each theme have been collated. | Yes |
|  | 5 | Themes have been checked against each other and back to the original data set. | Yes |
|  | 6 | Themes are internally coherent, consistent, and distinctive. | Yes and discussed with co-authors and PPI group. |
| Analysis | 7 | Data have been analysed- interpreted, made sense of- rather than just paraphrased or described. | Yes |
|  | 8 | Analysis and data match each other- the extracts illustrate the analytic claims. | Yes |
|  | 9 | Analysis tells a convincing and well-organised story about the data and topic. | Yes |
|  | 10 | A good balance between analytical narrative and illustrative extracts is provided. | Yes |
| Overall | 11 | Enough time has been allocated to complete all phases of the analysis adequately, without rushing a phase or giving it a once-over-lightly. | Yes |
| Written report | 12 | The assumptions about, and specific approach to, thematic analysis are clearly explicated. | Yes (see analysis process figure 1 and Supplementary file 1) |
|  | 13 | There is good fit between what you claim you do, and what you show you have done- i.e. described method and reported analysis are consistent. | Yes |
|  | 14 | The language and concepts used in the report are consistent with the epistemological position of the analysis. | Yes |
|  | 15 | The researcher is positioned as active in the research process; themes do not just ‘emerge’. | Yes (see positionality statement) |
