# Supplementary material for: Supported storytelling through the ‘Life Threads’ approach for family members after traumatic brain injury: “We’ve been through all of this trauma, and you’re giving me some string?”
Source: PLoS One. 2026 May 18;21(5):e0349304. doi: 10.1371/journal.pone.0349304 (PMC13183248; doi:10.1371/journal.pone.0349304)
Supplement: S2 Table — This is a completed checklist commensurate with reflexive TA to ensure all relevant methods are reported. (DOCX) [file pone.0349304.s002.docx]

**Supplementary Material Table 2: Reflexive Thematic Analysis Reporting Guidelines (RTARG)**

| Advice for aspects of the research report/approach to reporting | Guiding notes and further  explanation | Practices, concepts and  terminology to avoid | Response |
| --- | --- | --- | --- |
| The Introduction  NB: We prefer Introduction over Literature Review as a section heading, to capture the broader purpose of this section | | | Line 54 |
| Background and rationale | | | |
| Provide a robust context and rationale for the proposed research in the Introduction. | Can discuss existing research, theory, and the wider context; the researcher is understood as entering a conversation with existing scholarship. | Critiquing the methodological limitations of existing research from a (post)positivist/ quantitative standpoint; orienting a literature review to finding a “gap” that the research fills. | Lines 50-77 we discuss existing research and wider context of support for families after TBI we describe the background to the LTA and justify a qualitative investigation. |
| Clearly articulate a research question one that is methodologically coherent. | Can discuss refining an initially broader research question to a more specific one for the paper. | Formulating research questions as hypotheses or expectations about what might be “found”. | Line 78 research aim provided and situated within a qualitative pre-feasibility design. The aims and objectives are broad and exploratory. |
| “Owning your perspectives”^6^ | | | |
| Include information on guiding theoretical assumptions and other (e.g., explanatory) theory informing the use of TA. | Guiding (e.g., paradigmatic, ontological and epistemological) and other theory should be coherent with RTA. | (Post)positivism and (simple) realism. | Line 90 the study is clearly situated within an interpretivist paradigm with a relativist ontology and constructivist epistemology consistent with the narrative orientation of the LTA.  Line 183 the study is identified as inductive allowing the TA to be informed by the data and not one specific theory. |
| Report in a way that is consistent with stated theoretical assumptions throughout. | Theoretical coherence is evidenced through the use of language and concepts (e.g., around theme development,  research subjectivity, data interpretation), the treatment of data, and use of quality practices consistent with RTA. | Inadvertently “mashing-up” of RTA and (post)positivism/ realism (e.g., assuming data interpretation can be accurate and reliable) without a clear rationale. | Reporting maintains a commitment to relativism, honoring multiple realities and subjectivity of lived experience. |
| Evidence methodological coherence/ integrity in both the research and the report.^7^ | Theoretical assumptions, research questions, methods/practices of data generation, RTA, and specific orientation to RTA, purpose of research etc. all “fit together”, conceptually. | Ontological and epistemological confusion (e.g., claiming constructionism but focusing on lived experience and treating language as a transparent window onto this). | As a qualitative pre-feasibility study we have remained committed to the orientation of rTA whilst also investigating a complex intervention and answering questions about acceptability and perceived benefit. |
| Show evidence of reflexive practice. | Can discuss researcher professional or personal positioning and experience in relation to the topic, and/or participant group, and/or their role in shaping the research; use of reflexive journaling. | Evoking researcher bias (positivist), or even researcher influence, in a way that evokes it as possible rather than inevitable. | Line 165 positionality statement.  Supplementary Material 1 |
| Write in a methodologically coherent style. | A first-person writing style suits RTA, as it “writes in” the researcher and contributes to situated and reflexive reporting. | A third person writing style – writing the researcher out of the research. | We have written in first person where appropriate to enhance the situated and reflexive writing style required in rTA. |
| The Methodology  NB: We prefer the theoretically-embedded term Methodology as a section header, over the proceduralist term Method. | | | Line 89 |
| Participants/data items |  |  |  |
| Describe selection of participants/data items. | Should include criteria for selection and/or recruitment strategies and settings. | Terms “sample/sampling”, which connote “sampling” from a population (for the purpose of statistical generalisation). | Setting Line 119  Sample Line 124  Table 3 inclusion criteria |
| Describe number of participants/data items; provide a rationale or explanation around dataset or participant group size/composition. | Non-positivist qualitative concepts, such as “information power” or sufficiency offer conceptually appropriate justifications for “dataset” or “participant group” size and composition.^8^ | Justification based on saturation (simple realist), or statistical models (positivist); reporting rates of non-participation (an indicator of the representativeness of the “sample” in quantitative research). | Line 201  Protocol paper |
| Discuss characteristics of participants/ data items. | Balance the need to “situate the participant group” with participant anonymity (e.g., aggregate or report minimal demographics where appropriate).6 | Tables with each participant’s demographic information listed line-by-line. | Aggregate sample demographics Table 4 |
| Detail ethical approval and ethical code/principles followed, participant informed consent, etc. | Ethical discussion usually includes institutional ethical approval (if needed), but may include wider principles; providing research materials (participant information, consent form, etc.) in supplementary materials may be useful to support reflexive openness. | Compromising participant anonymity by the details provided. | Ethics Line 95 including details of informed consent, risks and mitigation.  Further details are provided in the protocol publication. |
| Dataset generation  NB: We prefer the term generation over collection to capture the active role of the researcher and that data don’t pre-exist research as data, but become data through research practices. | | | Line 142 |
| Provide some rationale for method(s) for data generation/data item sources chosen. | Discuss why the method(s) of data generation/data source was a good fit with the research question, participant group, guiding theory, etc. If multiple data sources are used, any rationale for combination should be conceptually appropriate (e.g., crystallisation^9^). | Triangulation as a rationale for different data sources (realist). | Line 143-160 dataset generation is described and justified as being a good fit to address the research objectives. |
| Describe development and/or characteristics of data generation tool(s). | Include tool(s) in supplementary materials when possible; discuss piloting if used, and any changes following piloting, or during data generation. | Using an existing tool with the aim of replicating existing “findings”, or developing and describing a tool in a way that is intended to facilitate future replication (positivist). | Interview and focus groups schedules are published as supplementary materials in the protocol paper. This is highlighted in the manuscript (Line 160) |
| Include details such as modality and/or setting of data generation, time frame, and other pertinent procedural information. | Relevant information includes: the mode of a data generation tool (e.g., video call focus groups; chat-based interviews); the context of data generation (location; timeframe) – where this doesn’t compromise participant anonymity; and mode of recording interactive data generation. | Standardisation as a gold standard (realist); justifying an aimed for standardisation in data generation tools as a means to facilitate the “reliability” or “accuracy” of the research; treating a lack of standardisation in data generation method, modality or setting as a problem, a potential source of “bias”. | Relevant information includes:  - Mode of data generation (online and in person)  - Location of in-person interviews.  - Method of recording. |
| Describe who conducted any interactive data generation (which author or research  role), and how. | Can include what, if anything,  the researcher \disclosed about  their personal or professional  positioning or motivation;  what skills and experience  they brought; note  researcher’s relationship with  participants prior to, during  and after the research. | Seeking standardisation (e.g.,  through the training of  researchers) in interactive data  collection; treating non- standardisation as a threat to  “reliability” or “accuracy”. | Authors involved in dataset generation referred to at relevant point using initials. Skills and experiences of authors is referred to in the positionality statement. |
| Describe the size/scope of dataset and dataset items. | Such as the range and average length for interviews/focus groups; range and average word length for textual data items. | Equating data quantity with data quality. | Timeframe (average and range) included for interviews and focus groups. |
| Describe, and if relevant explain, any preparation of data for analysis. | Such as method of transcription of audio/video data (a transcription key can go in supplementary materials); changes and “corrections” – such as why typographical errors in written data were corrected; system for removing any identifying information; use of pseudonyms and/or data codes. | Describing transcription as “verbatim” or “orthographic” with no further details; using edited or “cleaned up” data without acknowledgement of this; participant validation of the “accuracy” of transcripts (realist). | Line 182 – recordings were sent for professional transcription. Transcripts were checked for anonymisation and accuracy. |
| Data analysis |  |  |  |
| Provide some rationale for use of RTA, and, where relevant, for combining RTA with other approaches and procedures. | Any combining of RTA with other method/ologies or procedures should be warranted, rather than based on a misunderstanding of RTA, and conceptually coherent (unless clearly justified). | Citing generic characteristics of RTA (e.g., accessible, flexible) without explaining how they were relevant to the study; using a codebook without acknowledging this is not part of RTA and justifying its use. | Line 162  Further justification is included in the protocol paper p.8. |
| Describe specific orientation to RTA. | Locate RTA on dimensions of \inductive<>deductive and semantic<>latent. | A generic discussion of TA (or even RTA), not specifically situated in relation to the study or approach. | Line 185 an inductive approach was used.  Supplementary Material Table 1 – coding was both latent and semantic. |
| Discuss how the researcher(s) engaged with the analytic process. | Provide a specific and situated account of the analysis process; use supplementary materials to provide a fuller account of the analytic process. | Offering a generic description of the six phases of RTA in lieu of an account of analytic process. | Figure 1 and Supplementary Material Table 1 |
| Where more than one person is involved, describe who analysed the data (author or  research role). | Role(s) or involvement throughout the process should be discussed; where coding was collaborative, what this involved and how differences in coding and theme development were tackled, should be included. | Use of inter-coder agreement measures, consensus coding approach (positivist). | How each author contributed to the analysis is described in detail through Figure 1 and Supplementary Material Table 1 |
| Use language to describe the process and products of RTA that is coherent with the values and assumptions of RTA. | Language should convey the active role of the researcher(s) in “generating”, “crafting”, “constructing”, “creating”, “producing” or “developing” themes; language around themes should evokes them as products of a researcher-data process. | Passive language of discovery, such as “emerging”, “found”, “identified”, “discovered” – these evoke themes as “diamonds scattered in the sand” (p. 740)10; unexplained use of language and concepts from other approaches, such as emergent or superordinate themes (IPA), or line-by-line and/or open coding and constant comparison (grounded theory). | Appropriate use of active language is used to describe the analytical work in this study. Themes are situated as constructed through engagement with the data and our views and experiences as experts in this field. |
| The Analysis  NB: We prefer the heading Analysis over Findings/Results. Findings implies the researcher “found”, “discovered” or “identified” pre-existing themes. Results is strongly associated with the outputs of statistical analysis. | | | We prefer to follow convention for qualitative manuscripts and have chosen to keep the heading ‘findings’ in this manuscript. |
| Reporting the data analysis | | | |
| Provide an overview of themes or thematic structure. | Overviews can include a list, map or table of themes to preview the analysis. | An unclear thematic structure, including unexplained headings in the Analysis. | Table 7. Reflexive themes and associated sub-themes |
| Ensure theme conceptualisation is appropriate to RTA, and any divergences are justified and explained. | In RTA, themes report shared meaning, united around a central organising concept that differs for each theme. | Topic summaries; data generation questions reported as “themes”. | Each theme has a central organizing concept and these are described. |
| Name themes appropriately. | Use theme names that capture the “essence” or “story” of each themes; brief data quotations can be used. | (One-word) theme names that only identify a topic, and offer no story (evoking topic  summaries). | Themes have been discussed with co-authors and undergone several iterations to capture the essence of the theme. |
| Report themes in sufficient depth and detail. | As RTA is an interpretative method, themes should be multifaceted, and contain both data and analytic narrative; if useful, additional data extracts may be included in supplementary materials.^7^ | Thin, one dimensional themes, effectively conflating codes and themes; large number of themes relative to the length of the manuscript. | A substantial part of the manuscript has been devoted to reporting the themes in sufficient detail. |
| Use subtheme judiciously. | Themes are the main analytic purpose, and should be multifaceted; only use subthemes where doing so highlights an important facet or aspect of the central concept of a theme. | Fragmenting the analysis through overuse of subthemes, and an overly elaborated/“bitty” thematic structure. | Themes are accompanied by sub-themes to highlight important facets, each using a brief quotes give the sub-themes more communicative power. |
| Ensure the analytic narrative explains the meaning and significance of the data. | For RTA, each theme needs an analytic narrative that outlines its meaning and importance in relation to the topic, research question and dataset; the reader needs to be told about why/how data excerpts matter and “evidence” the theme; the Analysis section also needs to convey the overall story of the analysis. | Frequency counts as a justification for themes presented; simple paraphrasing of data as “analytic narrative”; treating data meaning as self-evident (data are assumed to speak for themselves); “arguing” with the data (treating the data as something to [dis]agree with, rather than to interpret and make sense of). | We have worked hard to ensure that each theme tells its own story in terms of how it contribute to addressing the study aims. |
| Provide an appropriate balance of analytic narrative and data extracts – both data extracts and analytic narrative  matter. | The rich descriptive and/or interpretative story of the analysis needs to be woven around sufficient analytic extracts from across the dataset. | Presenting either a long string of data extracts with barely any analytic narrative, or only the researcher’s narrative summary of the theme, without any data extracts to support it. | We have balanced the interpretive component of the findings with supporting data from a range of participants. |
| Demonstrate coherence between analytic narrative and illustrative/evidentiary data extracts. | Data extracts should convincingly and compellingly evidence the analytic claims | Mismatches between data extracts and analytic claims; not countering obvious alternative readings of the data. | Data extracts have been carefully chosen to evidence the analytical claims. |
| Integrate existing research and theory into the analytic narrative. | In RTA, an interpretative analytic narrative is enriched by incorporating relevant existing research and theory into the reporting of themes, reflecting notions of contextualised meaning, and contributing to an ongoing “conversation” about a topic. | The positivist tradition of separating a description of analytic “Results” and their interpretation with reference to scholarship and theory in a “Discussion” section. | We chose a more traditional approach and presented the findings without referring to existing theory. Theory is cited throughout the discussion. |
| The Final Section – A General Discussion or “Conclusions”  NB: We don’t have a preference for what a final section of an RTA report is called, and it depends on the context and the focus and purpose of the study – the heading Conclusion may evoke a certainty that isn’t appropriate; Implications may be useful; Final Considerations or Reflections may work, as might General Discussion. | | | Line 593 Discussion  We have also added a traditional conclusion Line 765 and feel this is an appropriate ending for this manuscript with messages for practice and future research. |
| Quality, evaluation and conclusions | | | |
| Draw analytic conclusions across themes. | Orient to the “so what” of the overall analysis – the “point” of the story told; this might include discussion of implications for practice and “actionable” outcomes.^11^ | Repetitive theme-by-theme integration of the analysis with existing literature; no overall conclusions drawn; no overall analytic story. | To draw conclusions across the themes the discussion is separated into four key points to convey the “so what” of the analysis. |
| Discuss implications or directions for future research. | Any suggestions for future research should stem from the analysis and be evidence-based (e.g., provide grounds for other groups potentially having different experiences or views) rather than generic. | Generic recommendations for other research, such as with a different “population”. | Future research priorities are summarized in Table 13. |
| Use and report quality practices coherent with RTA. | Ensure evaluation of research quality deploys conceptually coherent notions, such as: member reflections; crystallisation;^9^ others serving as a critical friend/sounding board to enhance insight;^12^ reflexive journaling. | Incoherent quality measures such as: member checking/participant validation; triangulation (realist); the use of theme agreement/consensus among researchers or corroboration of themes by another researcher (positivist). | Evaluation of the research is conceptually coherent and summarized in Supplementary Material Table 4. |
| Evaluate the research from a Big Q standpoint. | Such evaluation might including considering how the specifics of the study may have shaped the research produced (for example, the characteristics and context of the participant group/dataset; the methods and modalities for generating the data); situatedness should not be treated as a limitation. | Evaluations and descriptions of limitations that orient to quantitative or positivist norms, such as reference to lack of generalisability – positioned as a limitation, and equated only with statistical generalisability^13^ – or a “small” (by implication non-ideal) and “unrepresentative” “sample”. | S2 Response to Braun and Clarke’s (2022) 15-point checklist for ‘good’ reflexive TA |
| Include reflections on research process and practices, including researcher reflexivity. | Some consideration of the researcher(s)’s role in shaping the research and the knowledge generated is an important quality marker. | Reference to researcher bias/influence (positivist). | Positionality statement (Line 165)  Strengths and limitations Line 746 |
