# Supplementary material for: Supported storytelling through the ‘Life Threads’ approach for family members after traumatic brain injury: “We’ve been through all of this trauma, and you’re giving me some string?”
Source: PLoS One. 2026 May 18;21(5):e0349304. doi: 10.1371/journal.pone.0349304 (PMC13183248; doi:10.1371/journal.pone.0349304)
Supplement: S3 Table — This is a reflexive account of rigour and credibility in this study. (DOCX) [file pone.0349304.s003.docx]

**Supplementary Material Table 3. Response to Tracy’s big tent criteria**

| Criterion | Response |
| --- | --- |
| 1. Worthy topic | Given the complexity of family member’s experiences following familial TBI, lack of services and supportive ways to help family members understand the impact of injury on their own sense of self, this study was relevant, timely, significant and interesting. |
| 1. Rich rigor | This study used a multi-method design and facilitated rich engagement between participants and the study materials. CW met all participants prior to data collection and balanced online focus groups with in-person individual interviews that helped develop rapport and in-depth understanding.  Although the sample were homogeneous in terms of ethnicity, they were reflective of the heterogeneity of TBI including gender, relationship, age and time since injury. |
| 1. Sincerity | The study team adopted a reflexive approach and understood their own lens from which the study was conducted. Honesty and transparency were core team values, and we strived to be empathic, kind and self-aware in our interactions with participants and each other. |
| 1. Credibility | In this study credibility, or ‘trustworthiness’ was achieved through thick description and the exploration of different methods, different data and different researchers, allowed different facets to be explored, examined and challenged to deepen the analytical perspective gained through this research. |
| 1. Resonance | The final manuscript was written to achieve ‘empathetic validity’ which is the ability to facilitate an in-depth insight into the lived experience of the participants. This ability to affect the reader creates impact and the potential for the findings to be of value across a variety of contexts. |
| 1. Significant contribution | This study clearly extends our knowledge of both the impact of TBI on family members and if/how they may benefit from interventions that allow them to craft and own their own narrative. The power of this research was in its ability to make visible a hidden/ignored perspective and deepen understanding of this complex area. |
| 1. Ethics | Procedural ethics were always adhered to adopting safeguards for participants and the risk involved in this research. Methods such as unstructured interviews and inductive analysis are evidence of relational ethics through the mutual respect, dignity and connectedness between the researchers and the researched. Exiting ethics were also achieved through sharing research findings with participants and stakeholders and being open to continuing the dialogue about insights and understanding revealed through this research. |
| 1. Meaningful coherence | We are confident that the research achieved its stated purpose by directly responded to the research objectives. The study used methods and practices that were congruent with social constructivist positionality and acted within the commitments of a qualitative paradigm. Finally, we aimed to interconnect our findings with the current evidence base, demonstrating areas of convergence and revealing new insights. |
