# Supplementary material for: Supported storytelling through the ‘Life Threads’ approach for family members after traumatic brain injury: “We’ve been through all of this trauma, and you’re giving me some string?”
Source: PLoS One. 2026 May 18;21(5):e0349304. doi: 10.1371/journal.pone.0349304 (PMC13183248; doi:10.1371/journal.pone.0349304)
Supplement: Attachment — Submitted filename: Life Threads TBI Response to reviewers.docx [file pone.0349304.s004.docx]

We would like to thank the editor and both reviewers for their thoughtful and constructive feedback. We have carefully considered all comments and have revised the manuscript accordingly. Below we provide a detailed, point-by-point response.

Below are clarifications requested specifically by the Guest Editor.

| 1. Please ensure that your manuscript meets PLOS ONE's style requirements, including those for file naming. | We have now updated the main manuscript to align with style guides for the main body and title page.  Specific revisions include:   - Capitalisation removed from the title - Headers adjusted to required font size and style. - The inclusion of legends for the supporting information files   File names for the Supporting information have been changed to the required format of ‘S1_Table’ etc.  We note in the style guide for tables it states ‘Do not include graphic objects, images, or colored text’. The original manuscript included photographs alongside relevant direct quotes within tables. We have now removed these from tables and each image has been uploaded separately as a TIFF file. |
| --- | --- |
| 2. We note that the grant information you provided in the ‘Funding Information’ and ‘Financial Disclosure’ sections do not match. | I apologise for any discrepancies in the information provided. Unfortunately, I have been unable to identify the source of this discrepancy.  Under Financial Disclosure we state the required wording mandated by the funder  ‘This project is funded by the National Institute for Health and Care Research (NIHR) under its Research for Patient Benefit (RfPB) Programme (Grant Reference Number NIHR204092). The views expressed are those of the author(s) and not necessarily those of the NIHR or the Department of Health and Social Care.’  Under the current funding source we state ‘Research for Patient Benefit (RfPB) Programme’ alongside the Award Number NIHR204092.  We would be grateful if you could identify the sections that do not match and we will revise accordingly. |
| 3. Please state what role the funders took in the study.  If the funders had no role, please state: "The funders had no role in study design, data collection and analysis, decision to publish, or preparation of the manuscript." | I can confirm that the statement:  ‘The funders had no role in study design, data collection and analysis, decision to publish, or preparation of the manuscript.’  is correct |
| 4. Please revise your data statement to a non-author institutional point of contact. Please also include contact information for the third party organization, | The data statement has now been revised and contact information included.  Participants provided consent for their data to be used in future research. This will be decided on by the sponsor for researchers who meet the criteria for access to confidential data (nuhnt.researchsponsor@nhs.net). |
| …and please include the full citation of where the data can be found. | Not applicable. Data can only be accessed through the sponsor |
| 5. Can you please confirm that all participants gave consent for interview transcript to be published? | Participants **did not** consent for interview transcripts to be published. However, they consented to anonymised verbatim extracts of their transcripts to be published in the Informed Consent Form Point 6:  *The participant understands that anonymised quotes will be shared in publications arising from this study and that they will not be identified in these. They also understand that despite anonymisation that people close to them may still be able to recognise the information they have provided due to unique events and experiences they describe.* |
| If the participants DID NOT provide consent…  - Provide a de-identified version of the data or excerpts of interview responses.  - Provide information regarding how these transcripts can be accessed | All extracts used in the manuscript have been de-identified.  Further to this the interview transcripts have been de-identified prior to transferring these to the Sponsor. However, the transcripts still contain sensitive information and as such should not be in the public domain in full.  Participants did consent to future use in research. This will be decided on by the sponsor.  *ICF point 7: The participant understands that the information collected about them will be used to support other research in the future, and may be shared anonymously with other researchers.* |
| 6. If the reviewer comments include a recommendation to cite specific previously published works, please review and evaluate these publications | There were no such recommendations |

Please see our response to revisions requested by both reviewers

| Reviewer 1 | | |
| --- | --- | --- |
| The manuscript would benefit from a **clearer articulation** of the theoretical underpinnings of the Life Threads approach.  Referencing key frameworks (e.g., narrative identity reconstruction, post-traumatic growth, meaning-making) would strengthen conceptual grounding.  Clarify how the creative arts component (the “thread” metaphor) integrates with established psychological constructs to promote recovery and resilience. | Thank you for this helpful suggestion. We have now strengthen the manuscript by adding further explanation of theoretical underpinnings of the LTA including narrative, identity reconstruction and post-traumatic growth. Further, we have clarified the thread metaphor and how this integrates with creative arts approaches. | p.3-4 |
| Specify participant recruitment strategies and inclusion/exclusion criteria (e.g., relationship to TBI survivor, time since injury, level of functional impairment). | We have updated Table 3 to include the exclusion criterion for this study.  We have added further detail on the recruitment process to ensure this is transparent. I can confirm there were no restrictions based on injury type, family role or level of impairment. We have clarified this in the manuscript. | p.7 - 8 |
| Elaborate on data collection — for example, duration and format of sessions, interviewer background, and reflexivity considerations. | Under the guidance of Braun and Clarke, we use the header ‘dataset generation’ to refer to data collection. However, we acknowledge that it may not be immediately clear that dataset generation refers to data collection. We have therefore added ‘data collection’ to the heading for clarity.  We can confirm that all details pertaining to the duration and format of the interviews and focus groups are described here.  Interviewer backgrounds are contained within a ‘positionality statement’ under the main header of analysis. | p.9 |
| Describe the analytic process more thoroughly: How were themes generated, reviewed, and validated among authors? | Thank you for requesting further information on this important step. We have now moved the information from the supporting file into the main manuscript so that our methods are transparent and explicit for readers. | p.11-12 |
| Was data saturation achieved? | You are right to raise the question of data saturation. However, in the analytical method we have used, saturation is contentious and Braun and Clarke have written extensively about the lack of alignment of data saturation with their reflexive approach to thematic analysis.  We have now added clarity on this point to the manuscript and signposted to a more appropriate concept of ‘data sufficiency’. | p.8 |
| Consider including a brief reflexive statement addressing the researchers’ positions and potential influence on interpretation. | We do have a positionality statement that addresses all of the researchers’ backgrounds. I have now added to this that our experiences within this field will have influenced how we interpreted the data. | p.10 |
| Ensure each theme is consistently illustrated with verbatim excerpts from multiple participants. | I have mapped the respondent quotes against the themes and identified those where no data were presented.  I have now added data from Res 15, 16, 18 and 20.  The manuscript now contains data from every participant at least once. | Findings |
| In Table or Appendix form, provide a concise summary of major themes/subthemes and representative quotes to improve accessibility and transparency. | I can confirm that Table 8 provides a useful summary of the themes and sub-themes.  Direct quotes are contained in this table; however, these were not shown as such. We have now added quotation marks so that this is clear. | Table 8 |
| Clarify whether the quotes were edited for brevity or grammatical correction and how confidentiality was preserved. | Quotes used in the manuscript are de-identified verbatim extracts of raw data. We have now explained this in the manuscript. | p.16 |
| The discussion insightfully links findings to emotional adaptation and meaning-making, but could be deepened by connecting to broader literature on family identity reconstruction after TBI, ambiguous loss, and family systems recovery. | Many thanks for this important suggestion. We have now extended the debate around emotional adaptation and key constructs in the ABI literature including ambiguous loss and the family tasks model. We have also discussed the wider literature on family interventions after ABI (as reflected in a recent scoping review). | p.35-36  P.39-40  p.41 |
| Address possible limitations in generalizability, as participants may represent families with higher engagement in rehabilitation or more open communication styles. | Thank you for identifying this important limitation. We have now added this to the strengths and limitation section of the manuscript. | p.41 |
| The phrase in the title (“you’re giving me some string?”) powerfully captures ambivalence; consider analyzing this further as an example of the initial skepticism families may feel toward non-traditional interventions and how that evolves into acceptance. | Thank you for this helpful suggestion. We have now alluded to this initial doubt in under the sub-title. | p.34 |
| Expand briefly on ethical safeguards for emotionally sensitive interviews (e.g., availability of psychological support if participants became distressed). | Our ethical safeguards for participant’s emotional wellbeing are now described as requested. | p.5 |
| Include implications for clinical implementation — for instance, how Life Threads could be integrated into multidisciplinary neurorehabilitation settings or adapted for other caregiver populations. | We have made it clear that we are not ready to recommend use of the LTA in clinical practice without further research. However, we do advocate for family centred rehabilitation and that families have access to supportive interventions in their own right. We have also signposted to the Life Thread Network for people interested in the approach ([www.anchorpointabi.org/research](http://www.anchorpointabi.org/research)) | p.44 |
| The title is evocative and engaging; ensure quotation marks are consistent with journal style. | I have reviewed the style guides for manuscript titles. I can confirm that the title meets the character limit. There does not appear to be clear guidance on the use of quotation marks. However, we have revised the title to use quotation marks specifically. | p.1 |
| The abstract should explicitly mention sample size, analytic approach (e.g., reflexive thematic analysis), and main themes. | We have made revisions to the abstract to ensure these methods are explicit. | p.2 |
| Minor grammatical and stylistic adjustments are recommended (e.g., “effecting” → “affecting”). | We have reviewed the manuscript and made adjustments to improve grammar and correct errors. |  |
| References to similar narrative or arts-based rehabilitation programs (e.g., creative reminiscence therapy, photovoice) would enhance context. | Thank you for suggesting this. We have reflected on the basis of the approach in creative arts in the introduction and have returned to related work in the discussion. Here we also recognise the potential for methods originating as research methods (such as photo elicitation and the ‘wool and stones’ approach) in offering creative opportunities for resources or interventions to help families negotiate the challenges of life after brain injury and to find new and adaptive meanings. | Introduction/ Discussion |
| Verify formatting of quotations (indentation, punctuation) per PLOS ONE style guide. | I have reviewed the style guides for PLOS ONE and cannot see explicit guidance for the presentation of direct quotes. I have reviewed recently published qualitative manuscripts and there does not appear to be a consistent approach. The current manuscript is in line with current styles. |  |

| Reviewer 2 | | |
| --- | --- | --- |
| Re-title the manuscript as a "Qualitative Proof-of-Concept" rather than a "pre-feasibility" study, as your data concludes that a traditional randomized control trial (RCT) is likely inappropriate for this intervention. | Thank you for this suggestion which we have discussed at length. However, given that the published protocol and original funding were aligned to a qualitative pre-feasibility study we feel it is important to maintain this consistency. We do then highlight that the answers to the objectives were not as clear cut as those typically yielded in pre-feasibility studies in the strengths and limitations. | N/A |
| The sample is 95% White and 85% female; please expand the discussion on how the metaphor-heavy "Life Threads" approach might be received by more diverse cultural groups or male family members. | This is an important point and one that we explored in our PPIE group. We have added this insight into the manuscript. | p.44 |
| Explicitly address whether the approach can be a standalone tool, as the data indicates success relied heavily on skilled facilitation and expert-led unstructured interviews. | We have now added to Table 9, that we do not support use of the Life Threads approach as a standalone tool. | Table 9 |
| Briefly detail the vetting process used to manage the 22% of expressions of interest identified as phishing attempts to ensure data integrity. | Thank you for requesting clarification of this point. I have added further detail to the manuscript about this. | p.12 |
| Include any available insights into why three participants withdrew after the first focus group to better evaluate the intervention's "acceptability". | I have now added further insight into why these three family members felt unable to complete the LTA. However, this data was not collected formally. | p.12-13 |
| While the study follows Reflexive Thematic Analysis (rTA) standards, ensure the re-framing acknowledges the shift from quantitative feasibility to qualitative insight. | Thank you for this helpful recommendation. This has been added to the manuscript. | p.43 |
